# Extent of Spine Deformity Predicts Lung Growth and Function in Rabbit Model of Early Onset Scoliosis

**DOI:** 10.1371/journal.pone.0136941

**Published:** 2015-08-28

**Authors:** J. Casey Olson, Ayuko Takahashi, Michael P. Glotzbecker, Brian D. Snyder

**Affiliations:** 1 Center for Advanced Orthopaedic Studies, Beth Israel Deaconess Medical Center, Boston, Massachusetts, United States of America; 2 Department of Biomedical Engineering, Boston University, Boston, Massachusetts, United States of America; 3 Department of Orthopaedic Surgery, Boston Children's Hospital, Boston, Massachusetts, United States of America; Public Health Research Institute at RBHS, UNITED STATES

## Abstract

Early onset deformity of the spine and chest wall (initiated <8 years of age) is associated with increased morbidity at adulthood relative to adolescent onset deformity of comparable severity. Presumably, inhibition of thoracic growth during late stage alveolarization leads to an irreversible loss of pulmonary growth and thoracic function; however the natural history of this disease from onset to adulthood has not been well characterized. In this study we establish a rabbit model of early onset scoliosis to establish the extent that thoracic deformity affects structural and functional respiratory development. Using a surgical right unilateral rib-tethering procedure, rib fusion with early onset scoliosis was induced in 10 young New Zealand white rabbits (3 weeks old). Progression of spine deformity, functional residual capacity, total lung capacity, and lung mass was tracked through longitudinal breath-hold computed tomography imaging up to skeletal maturity (28 weeks old). Additionally at maturity forced vital capacity and regional specific volume were calculated as functional measurements and histo-morphometry performed with the radial alveolar count as a measure of acinar complexity. Data from tethered rib rabbits were compared to age matched healthy control rabbits (N = 8). Results show unilateral rib-tethering created a progressive spinal deformity ranging from 30° to 120° curvature, the severity of which was strongly associated with pulmonary growth and functional outcomes. At maturity rabbits with deformity greater than the median (55°) had decreased body weight (89%), right (59%) and left (86%) lung mass, right (74%) and left (69%) radial alveolar count, right lung volume at total lung capacity (60%), and forced vital capacity (75%). Early treatment of spinal deformity in children may prevent pulmonary complications in adulthood and these results provide a basis for the prediction of pulmonary development from thoracic structure. This model may also have future use as a platform to evaluate treatment effectiveness.

## Introduction

Lung growth is limited to the confines of the thoracic cavity comprised of the spine, rib cage, sternum and diaphragm. Airflow for respiratory function is driven by mechanical action of the rib cage and diaphragm. Congenital anomalies affecting growth and development of the spine and/or ribs may severely impact lung growth and respiratory performance. However, the relationship between such deformities to respiratory development is not well understood.

In humans, as growth proceeds, the increase in thoracic volume is asymptotic: at birth thoracic volume is 6.7% that of an adult, increasing to 30% by age 5 years, 50% by age 10 years, then doubling again by skeletal maturity [1, 2]. By contrast, the lungs increase in volume with alveolar multiplication up to 8 years of age [3], though this time point is controversial [4, 5], and after 8 years lung volume increases progressively via alveolar enlargement.

The relationship between development of the thorax, lungs and associated respiratory function is confirmed by congenital and childhood diseases that impose structural deformities of the spine and/or rib cage. For example, scoliosis, a lateral curvature of the spine that induces an associated deformity of the rib cage has dramatically different effects on respiratory function depending on the age of onset. Adolescent idiopathic scoliosis (AIS), which has onset relatively late during the growth of the lung and thorax (age >8 years), has less impact on long term respiratory function, compared to early onset scoliosis (EOS, age <8 years), which profoundly restricts lung development and respiratory function. In a retrospective study untreated EOS was associated with mortality 300% above normal from respiratory failure or cardiovascular disease, such an increase was not significant in adults with AIS [6]. Untreated EOS patient’s exhibit decreased vital capacity [7], and in limited post-mortem evaluations show hypoplastic alveolar development [8, 9]. However, studies have found a weak or non-existent correlation between severity of coronal plane deformity with respiratory function [7, 10, 11]. Campbell, et al. emphasized that in EOS the 3-dimensional structural malformation of the spine and ribs collectively affect the volume, symmetry, and function of the thorax and growth of the lungs [12].

The introduction of “growth-sparing” therapeutic strategies, which aim to correct spine and/or chest wall deformity without vertebral fusion have created a renewed interest in the natural progression of respiratory development in EOS. Such treatment has shown clear success in correcting deformity while maintaining normal rates of spine growth and by improving the respiratory status of critical patients [13]. However in reports of increased lung growth, body mass, and absolute vital capacity the extent of growth due to intervention cannot be determined due to the lack of an untreated control population for comparison and accordingly the goals of therapeutic intervention remain controversial.

The objective of this study is to develop a rabbit model of significant EOS through surgical unilateral rib-tethering of young rabbits, 3 weeks of age, and use this model in a controlled study evaluating respiratory growth and function to maturity, at 28 weeks of age. Through this model we were able to quantify growth and functional expectations through regular computed tomography scans and pulmonary function testing. We confirm the hypotheses that 1) limiting growth of the thorax during active development of the thorax and lungs will create structural deformities that provoke increased mechanical impediment to respiration and induce postnatal pulmonary hypoplasia, and 2) the extent of the 3-dimensional spine and thoracic deformity predicts respiratory growth and function at skeletal maturity.

## Methods

**Fig 1:**
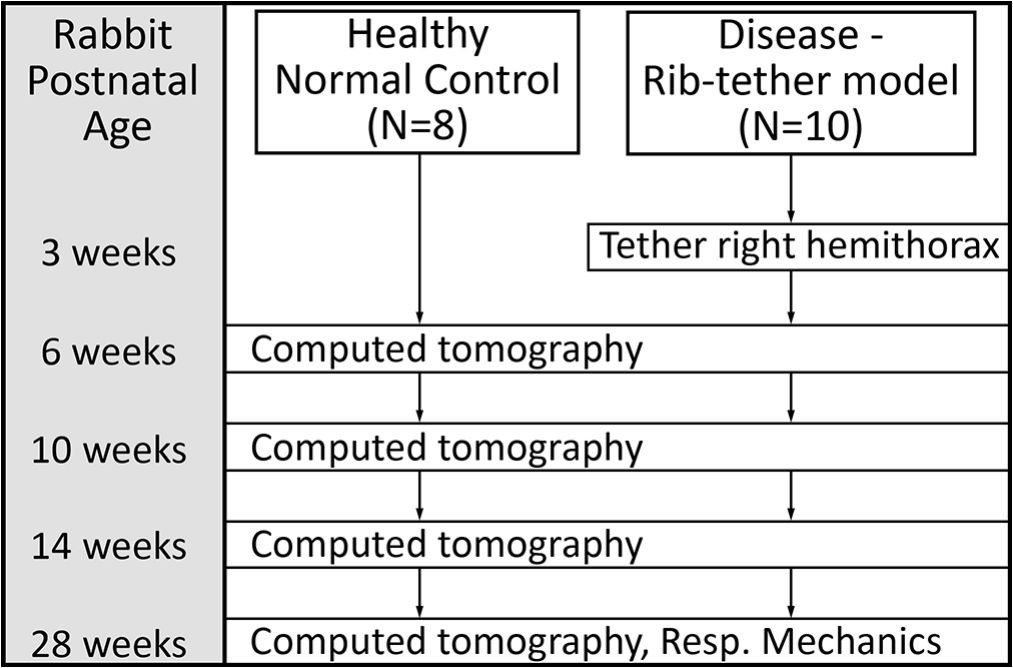
Study overview. Timing of surgery, CT scans, and respiratory mechanics testing relative to post-natal age of rabbits are indicated.

All animal procedures were approved and monitored by the BIDMC institutional animal care and use committee. Rib-tethering surgery to induce EOS was performed as soon as the rabbits could be weaned from their mother at age 3 weeks (Fig 1). Based on somatic growth of the rabbit compared to the human, a 3 week old rabbit is equivalent to a 3 year old child, while a 28 week old rabbit is considered equivalent to a full grown adult. Pulmonary growth (alveolar septation) in the rabbit is thought to continue until skeletal maturity, with the rate of growth decreasing monotonically from the first day of birth [14]. Power analysis based on preliminary data [15, 16] from pilot studies indicated that 8 rabbits per group were required to detect a 20% mean difference in respiratory compliance and lung volume between healthy control rabbits (Normal) and unilateral tethered-rib rabbits (Disease). All data files are available from the Harvard dataverse database (accessible at https://dataverse.harvard.edu/dataverse/REOS).

### Rib-tethering surgery

**Fig 2:**
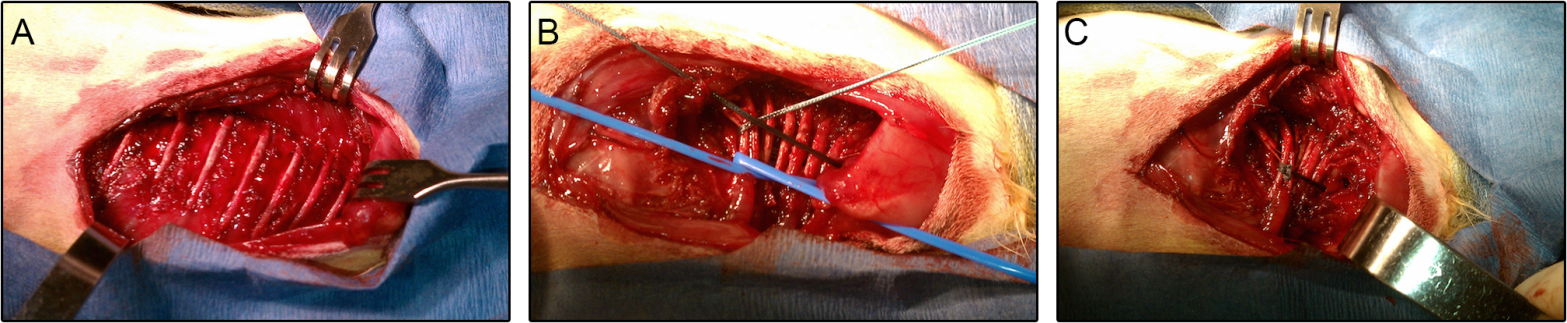
Tethering surgery. Tethering ribs 3–9 comprising the right hemithorax: (A) longitudinal incision exposing ribs; (B) ribs constricted with blue vessel loop; (C) polyester suture tied off to maintain constriction of right hemithorax, vessel loop removed.

At 3 weeks of age, ribs 3 through 9 comprising the right hemithorax were tethered to create a progressive, convex left, kypho-scoliosis. Rabbits were anesthetized with Ketamine (35 mg/kg) and Xylazine (2.5–5 mg/kg) administered intramuscularly, intubated and placed on ventilator control with isoflurane gas. The rabbits were positioned prone and the right chest sterilely prepped. A longitudinal incision through the skin was made along the posterior angle of ribs 2–10 and then deepened sharply to the intercostal muscles. Ribs 3–9 were exposed by sharp dissection (Fig 2A), the overlying periosteum incised, and each rib elevated from its periosteal bed, preserving the integrity of the underlying parietal pleura. An elastic vessel loop was passed under all the ribs and tightened to constrict the right hemithorax (Fig 2B). The tethered ribs were secured with a figure of eight #0 polyester suture (Fig 2C). The incision was closed in layers and the rabbits allowed to recover in a high-oxygen environment with a trans-thoracic tube for 24 hours in case of pnuemothorax. Post-operative pain was managed with Buprenex (0.03 mg/kg) immediately after surgery and Meloxicam (0.2 mg/kg) twice over the next 24 hours. Rabbits were visited daily to ensure they did not exhibit signs of pain or distress.

### Computed tomography imaging and analysis

To capture the asymptotic growth curve of the rabbit through skeletal maturity, serial CT scans of the entire thorax were obtained at post-natal ages 6, 10, 14, and 28 weeks. After intubation with a cuffed endotracheal tube and anesthetic induction with isoflurane, the rabbits were mechanically ventilated and positioned prone on a Toshiba Aquilion 64 scanner with resolution of 0.26x0.26 mm^2^ in the transverse plane, and 0.3 mm slice spacing. After applying a sequence of deep passive inflations to recruit regions of atelectasis and to induce a brief period of apnea, a breath hold lasting 3–5 seconds at constant airway pressure was maintained while the entire thorax was imaged. This procedure was performed four times at incremental inflation pressures: 0→5→15→25 cmH_2_O.

#### Spinal deformity

The degree of scoliosis was measured as the Cobb angle on the coronal plane CT projection (θ_S_) and the degree of kyphosis as the Cobb angle on the sagittal plane CT projection (θ_K_). The maximal spine deformity angle was estimated, $\theta_{M}=2*{\text{tan}}^{-1}\sqrt{{\text{tan}}^{2}(\theta_{S}/2)+{\text{tan}}^{2}(\theta_{K}/2})$, and reported (see S1 Appendix for derivation). Following clinical EOS classifications, rabbits with a maximal spine deformity >55° at 10 weeks of age were identified as having severe spinal deformity and those with maximal spine deformity <55° were identified as having moderate spinal deformity.

The thoracic rotation angle (TRA), the maximal thoracic rotation in the trans-axial plane, was measured at the apex of the spine deformity [12].

#### Calculation of aerated lung volume and mass

Lung mass, aerated lung volume, and fractional tissue volume (FTV) were calculated in each CT image for the left, right, and total lung [17]. The X-ray attenuation of each voxel in the CT image, represented by the Hounsfield unit (HU), is calibrated to tissue density (μ = 1 + HU/1000). Assuming that lung parenchyma has the density of water (μ = 1 g/mL) and the air filling the alveoli has negligible density (μ = 0 mg/mL), the lung mass was calculated as the sum: $m_{tissue}=\sum_{n=1}^{N}\left(1+{HU}_{n}/1000\right)\;*\;V_{pixel}$, and the aerated lung volume was calculated as the sum: $V_{air}=\sum_{n=1}^{N}\left(-{HU}_{n}/1000\right)\;*\;V_{pixel}$, of all voxels, *N*, contained within the segmented lung profile. The aerated lung volume at a tracheal breath hold pressure of 0 cmH_2_O estimates FRC, while the aerated lung volume at a tracheal breath hold pressure of 25 cmH_2_O estimates TLC. Inspiratory capacity is the difference between the two (IC = TLC—FRC). Fractional tissue volume (FTV) is then defined at TLC as the ratio of tissue volume over total lung volume: FTV = *V*_*tissue*_/(*V*_*tissue*_ + *V*_*air*_) where V_tissue_ has 1:1 equivalency to m_tissue_ in ml and grams respectively.

#### Analysis of local specific volume

**Fig 3:**
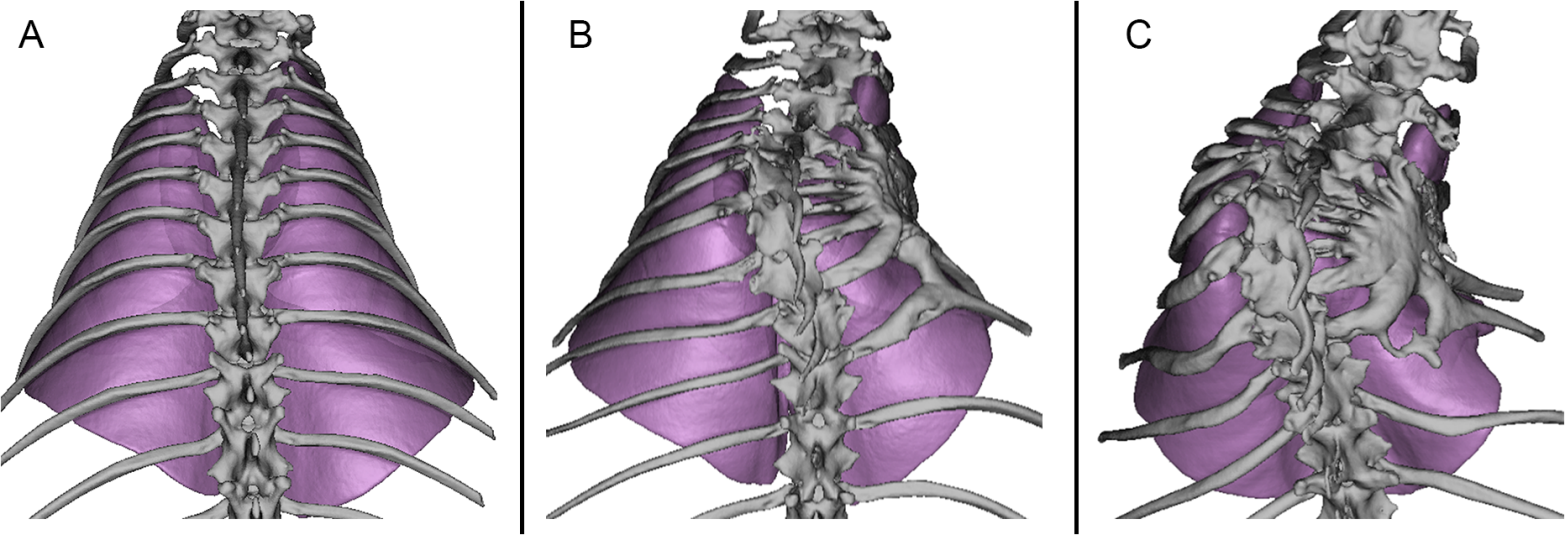
CT image reconstructions. Reconstructions are from 28 week post-natal age demonstrating deformity of thorax and lungs of a representative (A) Normal, (B) Moderate, and (C) Severe rabbit. The inferior surface of the lungs is colored orange.

To evaluate how thoracic deformity affected regional lung mechanics, the regional deformation of the lung parenchyma was measured in adult rabbits (28 weeks) as the lung was inflated from 0→5, 5→15, and 15→25 cmH_2_O tracheal breath hold pressure. Deformable image registration (DIR) was performed on each sequential set of incremental breath hold CT images using custom software to track voxel by voxel trajectories [18]. The local specific volume (sVol), was calculated from the Jacobian (*J*) of the local voxel based trajectories, $\text{sVol}=\frac{V_{h}-V_{l}}{V_{l}}=J-1$, and represents intrinsic volume expansion. Here *V*_*l*_ is the regional volume (i.e. voxel volume) at the lower breath hold pressure and *V*_*h*_ the volume occupied by that tissue at a higher pressure, calculated within each set as $\frac{V_{5}-V_{0}}{V_{0}},\;\frac{V_{15}-V_{5}}{V_{5}}$, and $\frac{V_{25}-V_{15}}{V_{15}}$ respectively. DIR results were validated by comparing the total change in the volume of the lungs measured on segmented CT images to the change in volume calculated from the *sVol* across the entire lung (Fig 3).

### Respiratory Mechanics

Respiratory elastance was determined at 28 weeks by fitting the ubiquitous single compartment model to the pressure-volume ventilation signal of anesthetized, prone positioned, and ventilated rabbits [19]. Briefly, 3 deep inspiratory maneuvers were performed on the ventilated rabbit to recruit regions of atelectasis after which pressure and flow at the airway was recorded over 3 consecutive ventilation cycles. Rabbits were ventilated at 24 breaths per minute with maximal inspiratory pressure (MIP) between 12 and 14 cmH_2_O tracheal pressures. Rabbits with ventilation signals acquired with MIP greater than 14 cmH_2_O were dropped from analysis as this inflated the respiratory system past the linear elastic limit of normal tidal breathing. Mean elastance and resistance values were determined with the recursive least squares method of Bates and Lauzon [20].

Forced vital capacity (FVC) was determined using the raised volume rapid thoracoabdominal compression technique. While the rabbits were intubated and deeply anesthetized, 3 deep inflations were provided prior to inflating the lungs to 25 cmH_2_O inspiratory pressure. A circumferential air bladder loosely fit around the thorax and abdomen was rapidly pressurized to 60 cmH_2_O and held until air ceased to flow from the endotracheal tube. The volume of expired air was integrated from the flow signal at the airway opening. This procedure was repeated several times to ensure reproducibility. After at least 2 quality expirations, the highest expired volume was recorded as FVC, but only if all measures were within a 10% disparity margin as described by ATS/ERS guidelines [21].

### Histology

After mechanical testing at 28 weeks all rabbits were sacrificed with pentobarbital during a median sternotomy, lungs and heart were then excised and suspended with 25 cm formalin pressure through the trachea maintained for 24 hours. Transverse oriented samples were taken of the right upper, right lower, left upper, and left lower lobes and embedded in paraffin. Two H&E sections were prepared from each sample, separated by 150 μm, and the radial alveolar count (RAC) performed on all suitable respiratory bronchioles as has been described to quantify pulmonary acinar development [22]. All slides were blinded and counts were performed by one observer (J.C.O.). Counts were averaged for each rabbit for the left lung, right lung and total lung.

### Statistical evaluation

Thoracic deformity, lung growth, and respiratory mechanics measures were compared among all groups of rabbits over time (Severe deformity, Moderate deformity, and Normal control) using two-way, repeated measures ANOVA with Bonferroni post-hoc analysis. One-way ANOVA was used on measures made only at 28 weeks. All rib-tether rabbits were pooled and linear regression was performed between the maximal deformity angle, *θ*_*M*_, at a post-natal age of 6 weeks against measures of respiratory mechanics for adult rabbits at 28 weeks. The zero-order Pearson coefficient (r) and coefficients of determination (R^2^) are presented. Statistical significance at p<0.05 is indicated.

## Results

### Thoracic and pulmonary development

Tethering the ribs comprising the right hemithorax immediately induced a significant convex left scoliosis (θ_S_ >40°) as measured post-operatively with fluoroscopy. However, during the rib tethering, several of the rabbits sustained rib fractures, which reduced the constrictive force of the tether and likewise the severity of the resultant spinal deformity, thus increasing variance in the Disease group and reducing statistical power. To increase statistical power, 2 additional Disease rabbits were added for a total of 10 Disease rabbits. Accordingly Disease rabbits were separated into Severe (θ_M_ >55°, N = 5) and Moderate (θ_M_ <55°, N = 5) deformity groups (Fig 3).

**Fig 4:**
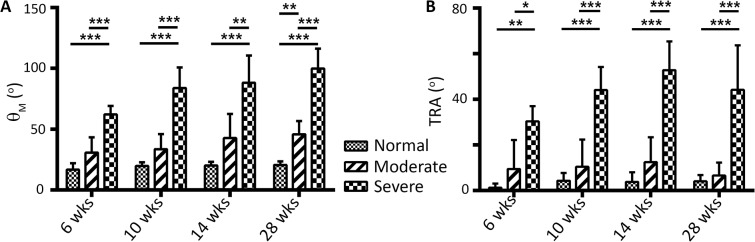
Deformity. (A) Maximal overall spine deformity, θ_M_, in each group at all time points and (B) TRA in each group for all time points are shown. Bonferroni statistical significance is indicated: *p<0.05; **p<0.01; ***p<0.001.

**Fig 5:**
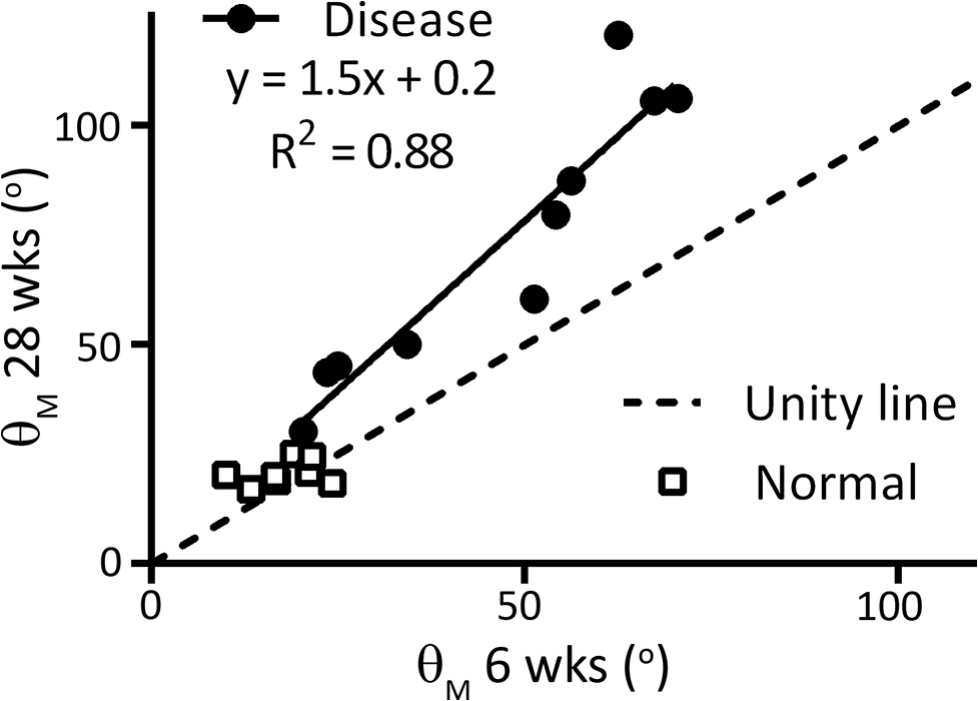
Progression of Deformity. Linear regression comparing spine deformity at 6 weeks to 28 weeks post-natal age is shown.

Compared to Normal, the Severe group had a significant spine deformity angle, θ_M_, (Fig 4A) and TRA (Fig 4B) by 6 weeks of age that progressed with growth, whereas the Moderate group achieved a significant θ_M_ only at 28 weeks. The θ_M_ at 6 weeks post-natal age was highly correlated in Disease rabbits (R^2^ = 0.88, p<0.001) to θ_M_ at adulthood, 28 weeks postnatal age and the linear regression slope was significantly greater than unity (p<0.05, Fig 5). There was also strong correlation between 6 and 28 weeks as measured by the kyphosis Cobb angle, θ_K_ (R^2^ = 0.79, p<0.05), but not the scoliosis Cobb angle, θ_S_ (R^2^ = 0.20).

Somatic growth was significantly reduced in rabbits with severe spine deformity by 6 weeks and at 28 weeks mean body weight in the Severe group was 89% +/- 5% of Normal (p<0.001).

**Fig 6:**
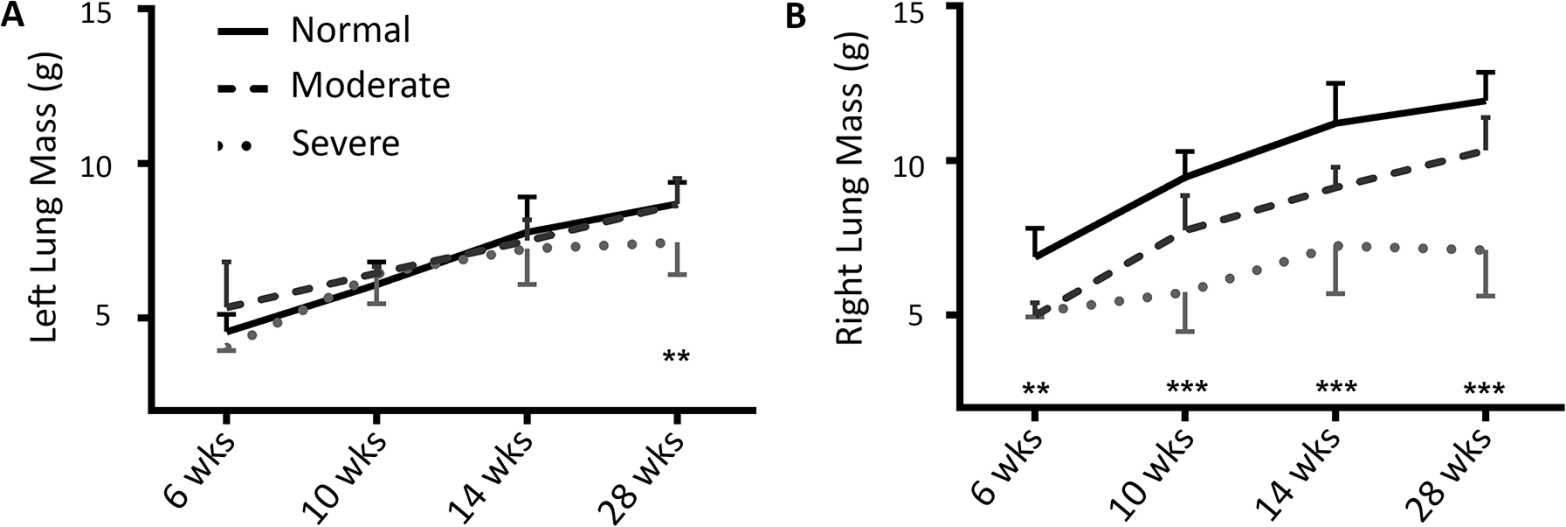
Lung mass. (A) Left and (B) right lungs mass at each time point is shown. Statistical significance is indicated: **p<0.01, ***p<0.001.

Volume and mass of the segmented left and right lungs was evaluated serially during growth (mass is shown in Fig 6); by 28 weeks, the total lung, right lung, and left lung mass in the Severe group was 71% +/- 12%, (p<0.001), 59% +/- 12% (p<0.001), and 86% +/- 12% that of Normal respectively; the corresponding lung aerated volumes at TLC were 79% +/- 10%,(p<0.01), 60%+/- 10% (p<0.001), and 105% +/- 14% of Normal. For the Moderate group the total, right, and left lung mass at 28 weeks was 93% (SD 9%, p<0.05), 87% +/- 9% (p<0.05), and 100% +/- 10% of Normal respectively; corresponding aerated lung volumes at TLC were 92% +/- 7%, 79% +/- 7% (p<0.05), and 108% +/- 8% of Normal. IC was affected in similar proportions to TLC (Table 1). FRC was reduced in the right lungs and increased in the left lungs on average in Moderate and Severe groups, however this measure was highly variable and not significantly different than Normal (Table 1).

**Table 1:** Comparisons of CT and histology derived measures of lung development in the left and right lungs.

	Left Lung		Right Lung	
	Mod.	Severe	Mod.	Severe
Mass	100% (10)	86% (12)	**87% (9)** *	**59% (12)** ***
FRC	137% (53)	150% (41)	68% (42)	63% (15)
TLC	108% (8)	105% (14)	**79% (7)** *	**60% (10)** ***
IC	98% (15)	91% (15)	80% (16)	**58% (12)** ***
FTV	93% (5)	84% (12)	106% (3)	98% (12)
RAC	90% (7)	**69% (12)** **	96% (7)	**74% (15)** **

Values are shown for each experimental group as a percentage of Normal, with standard deviation in parentheses. Statistically significant correlations are indicated:

*p<0.05

**p<0.01

***p<0.001.

The right to left lung volume ratio at TLC and 28 weeks was less than Normal (1.38 +/- 0.11) in the Moderate (1.03 +/- 0.07) and Severe (0.81 +/- 0.10) groups at all time-points (p<0.001).

FTV of the left lung was less than Normal in the Severe (84%, +/- 12%) and Moderate groups (93% +/- 5%) by 28 weeks, statistical differences were not detected. The ratio of right to left lung FTV at 28 weeks was greater than Normal (1.00 +/- 0.03) in the Moderate (1.15 +/- 0.04) and Severe (1.18 +/- 0.09) rabbits at 14 and 28 weeks (p<0.01).

### Respiratory Mechanics

FVC of one rabbit in the Severe group and three rabbits in the Moderate group had >10% disparity between repeated tests and were therefore removed from analysis. From the remaining rabbits FVC in the Severe group was 75% +/- 12% that of Normal (p<0.05). Differences in FVC between the Moderate group and Normal were not detected.

**Fig 7:**
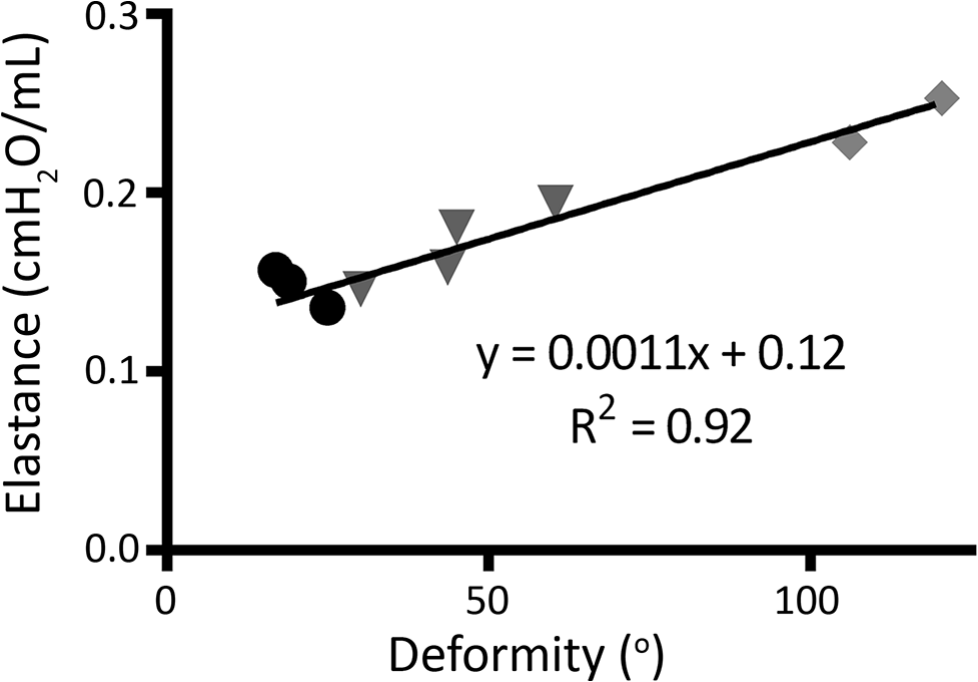
Respiratory elastance. Dynamic elastance versus spine deformity, θ_M._ Normal rabbits are indicated by circles, Moderate deformity by triangles, and Severe deformity by diamonds.

Respiratory elastance was compared in only 3 Normal, 4 Moderate, and 2 Severe rabbits, all other rabbits were ventilated with an MIP greater than 14 cmH_2_O and were dropped from analysis as this inflated the respiratory system past the linear elastic limit of normal tidal breathing. Respiratory elastance was increased in Severe and Moderate groups compared to Normal although significance was not found. However, elastance correlated very strongly with spine deformity angle (R^2^ = 0.92, p<0.001, Fig 7). No differences in resistance were noted.

**Fig 8:**
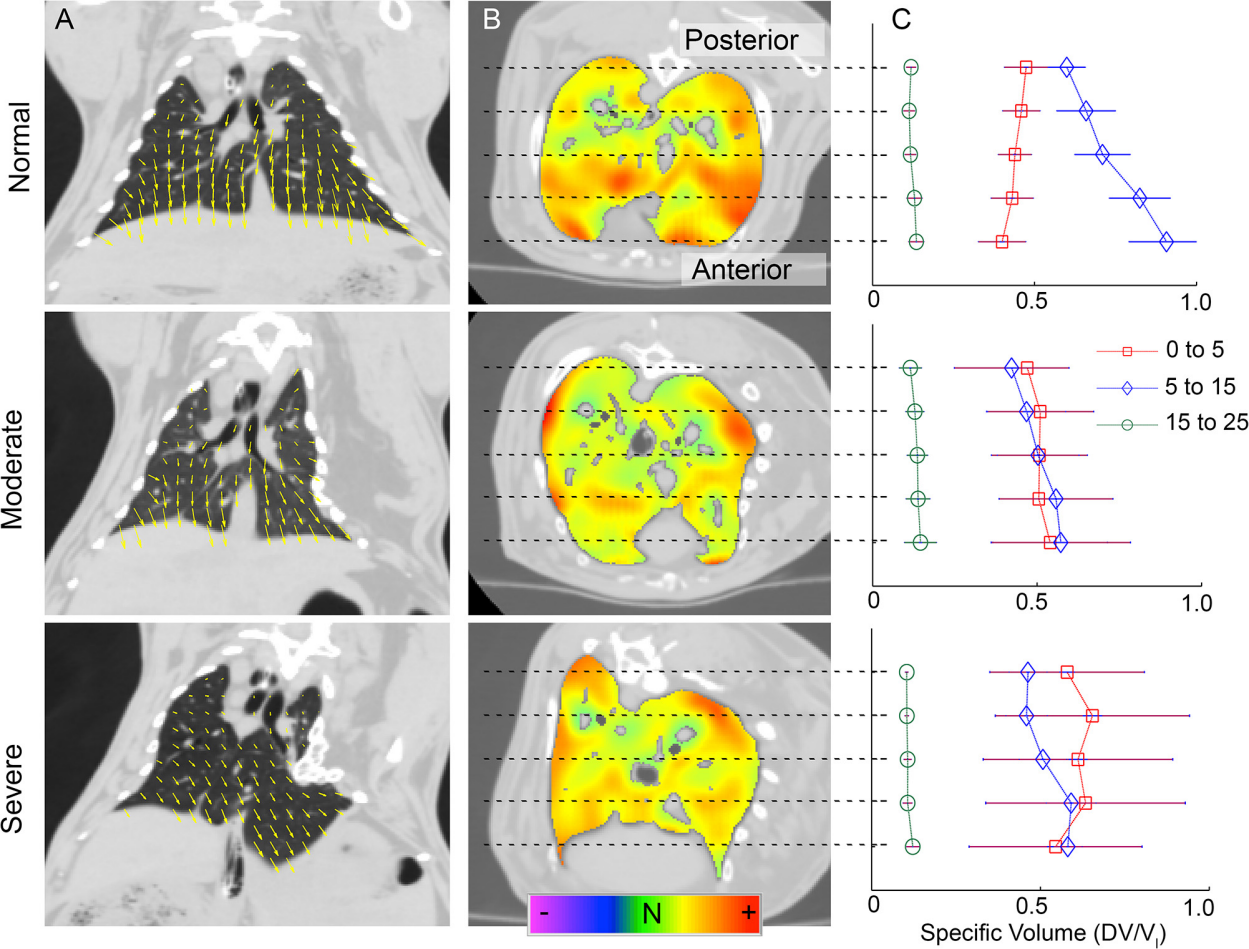
Local specific volume. Comparison of image registration results between Normal (top row), Moderate (center) and Severe (bottom) rabbits. Column (A): coronal slice with the registration displacement map, magnitude and direction of displacements are indicated by yellow vectors. Column (B): axial slice with derived sVol map, hot colors indicate greater expansion and green neutral. Column (C): Comparison of regional sVol, posterior to anterior, between Normal, Severe, and Moderate rabbits at each phase of inspiration. Error bars show SD.

Representative images of regional pulmonary displacement and sVol as respiratory pressure is increased from 5 to 15 cmH_2_O are shown in Fig 8A and 8B. At this phase, mid-inspiration, the anterior gravity-dependent lung of all rabbits shows increased or near even sVol compared to the posterior lung. In the early-inspiration phase (0 to 5 cmH_2_O respiratory pressure) the opposite is apparent (posterior>anterior) in all Normal rabbits but in no Severe rabbits (Fig 8C), here the ratio of posterior to anterior lung sVol in the Severe group is significantly greater than Normal (p<0.05). These differences in the posterior to anterior gradient in lung expansion are maintained through analysis of both the left and right lung individually. However the mean sVol was significantly less in the left lung compared to the right in disease rabbits at initial, mid, and end inspiration by 92%, 89%, and 85% respectively. There was no difference between the lung apex and base.

### Histology

**Fig 9:**
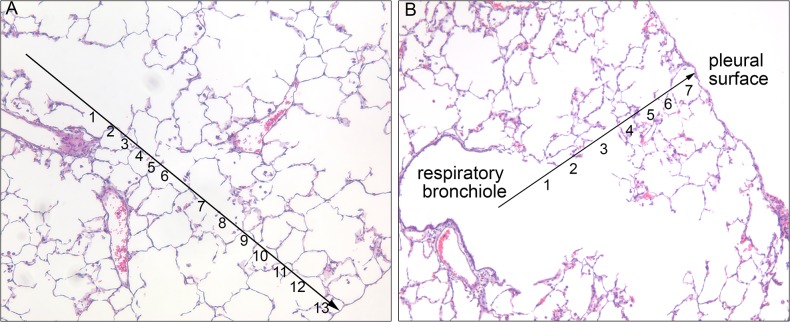
Radial alveolar count. Alveolar histology of a representative (A) Normal and (B) Severe rabbit showing the radial alveolar count. A line is drawn from the center of the respiratory bronchiole perpendicular to the nearest interlobular septa, each saccule bisected by this line is counted.

There was no indication of pulmonary edema or congestion in any slides as might be indicated by hemosiderin or an alveolar transudate. Average number of usable RAC fields per rabbit was 25.1, ranging from 7 to 42 (Fig 9), all fields were adjacent to the pleural surface. Total lung RAC in Severe rabbits (8.8 +/- 1.5) was significantly less than Moderate (11.4 +/- 0.8, p<0.01) and Normal (12.0 +/- 0.9, p<0.001). Similar results are seen for the left and right lung individually (Table 1). Total lung RAC for all rabbits strongly correlated with the CT derived total lung mass (R^2^ = 0.67, p<0.001).

### Correlation of structure with growth and function

Correlation of maximal deformity angle, θ_M_, at 6 and 28 weeks to growth and functional outcomes at 28 weeks are shown in Table 2 for Disease rabbits. Increased deformity angle at youth predicted reduced body mass, thoracic asymmetry, reduced TLC and IC due to right lung volumes, reduced lung mass in both lungs, reduced FTV in the left lung accordingly, and reduced RAC in both lungs. This also predicted a reduction in FVC and increase in dynamic elastance. Correlations to deformity at 28 weeks were similar and generally stronger.

**Table 2:** Correlation between maximal spine deformity with growth and functional outcomes in Disease rabbits.

Outcomes (28 wks)↓	Spine deformity, θ_M_	
	at 6 wks	at 28 wks
Body mass	(-) **0.43** **	(-) **0.46** **
**Asymmetry**		
R:L vol. ratio	(-) **0.65** ***	(-) **0.78** ***
TRA	(+) **0.70** ***	(+) **0.75** ***
**Lung Vol.**		
FRC	(-) 0.00	(+) 0.00
- Left	(+) 0.18	(+) **0.26** *
- Right	(-) 0.17	(-) 0.17
TLC	(-) **0.58** **	(-) **0.52** **
- Left	(-) 0.00	(+) 0.00
- Right	(-) **0.72** ***	(-) **0.76** ***
IC	(-) **0.58** **	(-) **0.53** ***
- Left	(-) 0.19	(-) 0.11
- Right	(-) **0.69** ***	(-) **0.75** ***
**Lung Growth**		
Mass	(-) **0.81** ***	(-) **0.87** ***
- Left	(-) **0.47** **	(-) **0.49** **
- Right	(-) **0.86** ***	(-) **0.95** ***
FTV	(-) 0.24	(-) **0.31** *
- Left	(-) **0.43** **	(-) **0.52** ***
- Right	(-) 0.03	(-) 0.04
RAC	(-) **0.65** **	(-) **0.76** ***
- Left	(-) **0.62** **	(-) **0.78** ***
- Right	(-) **0.63** **	(-) **0.69** **
**Resp. Mech.**		
FVC	(-) **0.50** **	(-) **0.64** **
Elastance	(+) **0.85** ***	(+) **0.92** ***

The coefficient of determination (R^2^) is shown with the direction of the correlation relationship in parentheses (+ or-). R:L.–right to left volume ratio. Statistically significant correlations are indicated:

*p<0.05

**p<0.01

***p<0.001.

## Discussion

The objective of this study was to create an animal model that develops severe thoracic deformity early in life to evaluate the relation between thoracic growth to growth and function of the lungs. Therefore we tethered the right rib cage of 3-week-old rabbits to provoke progressive constriction of the thorax during skeletal growth, limit the space available for the lungs to grow, distort the spine and diaphragm, and alter chest wall compliance. We tested the hypotheses: 1) structural deformities that limit the growth of the thorax during development of the lung, spine and rib cage increase mechanical impediment of respiration and induce postnatal pulmonary hypoplasia at skeletal maturity; 2) the extent of the 3-dimensional deformity of the spine and thorax during growth predicts pulmonary growth and respiratory function at skeletal maturity.

In support of hypothesis 1 we demonstrated that deformities of the spine and thorax acquired at an early age mechanically inhibit lung growth and restrict respiratory function. Lung mass, TLC, and FVC was noticeably reduced in rabbits with Severe deformity, while rabbits with Moderate deformity showed a lesser decrease in lung mass and volume, but no discernible impairment in respiratory function. To our knowledge this is the first animal model to create an early onset deformity of the spine and thorax for the purpose of serially evaluating pulmonary growth and function through maturity. The current rabbit model is an improvement over an initial attempt to create a rabbit model for scoliosis, in which a mild scoliosis (14°) was created without any significant impairment of lung growth [16].

In support of hypothesis 2 we demonstrated how the extent of spine deformity present in young, growing animals (6 weeks of age) is predictive of thoracic deformity and impaired pulmonary growth and respiratory mechanics at maturity (28 weeks). The maximal spinal curvature, θ_M_, measured in the young rabbits was predictive of abnormal spine deformity, body weight, lung mass, acinar development, TLC, respiratory elastance, and FVC at maturity. These particular outcomes may be targets for improvement in future controlled studies on effects of surgical intervention using this model; they may also translate to measures of clinical interest. In longitudinal clinical studies only FVC, measured at or near maturity, has thus far been shown to correlate with spine deformity measured in young children with EOS [7].

In our rib-tether rabbits, growth of the right lung, ipsilateral to the rib fusion, was clearly compromised in proportion to severity of the thoracic deformity. In the left lung, contralateral to the rib fusion, rabbits had a slight increase in volume such that in Moderate deformity rabbits (θ_M_ <55°) total lung volume was close to normal, however Severe deformity rabbits (θ_M_ >55°) exhibited a significant reduction in left lung mass and RAC, and similarly in FTV relative to the right lung, this indicates hyperinflation of generally underdeveloped acini in proportion to severity of deformity. RAC and lung mass together are reliable predictors of pulmonary hypoplasia; RAC is shown to correlate with lung mass under several conditions and with total alveolar number [22, 23]. Other investigators have correlated CT attenuation (FTV) in the lungs with alveolar septal density histologically in the evaluation of compensatory pulmonary growth in a dog model of pulmonary resection [24], here increased septation was found in the remaining lung as it expanded to fill the void, the authors suggested that increased strain in the remaining lung after resection [25] mechanically promoted alveolar septation [26, 27]. Similarly in our study the reduced sVol in the left lung compared to the right implies that this lung experiences reduced mechanical strain during normal respiration thus reducing alveolar septation through growth and the observed restraint in development of the left lung even though left lung volumes were near Normal.

CT non-rigid registration allows determination of the local expansion of lung parenchyma over the course of ventilation [28], as estimated here over static perturbations. The sVol map identifies the proportion of alveoli recruited during ventilation [29] and has been used to identify patterns of heterogeneous lung expansion in animal models of acute lung injury [30, 31]. Using image registration, we demonstrated that for a Normal rabbit in the prone position sVol varies according to height (anterior to posterior position) and inspiratory pressure. At initial-inflation (0 to 5cmH_2_O), alveolar recruitment occurs primarily in the posterior lung, while at mid-inflation (5 to 15cmH_2_O), the anterior portion of the lung plays a greater role (anterior > posterior). At large inflation pressures (15 to 25cmH_2_O), sVol is uniform from anterior to posterior at a decreased magnitude since the respiratory system is approaching its elastic limit. Therefore the regional variation in sVol as a function of height and inspiratory pressure implies that the intrinsic mechanical properties of the lung and thorax passively control the distribution of airflow that account for this regional variation in lung expansion. Moreover, this differential pattern of gravity dependent regional lung expansion is not apparent in the deformed rabbits. This finding suggests that in the Normal rabbit, the dependent portion of the lung contributes relatively more to pulmonary reserve capacity (recruited lung) and that this reserve capacity is diminished by thoracic deformity.

This study has a few limitations. The rib-tethering surgery was variably effective in creating thoracic deformity, in particular if there was an intraoperative fracture of the ribs during the tethering procedure, which reduced the deforming moment on the thoracic spine. The RAC metric used here has a known fixation inflation bias and may be susceptible to orientation bias in this disease model as only transverse samples were evaluated, a thorough and non-biased approach to histo-morphometry would provide stronger evidence on the process of lung development in this disease [32]. Additionally physiologic respiration was not quantified such as through acute and chronic decreases in the partial pressure of oxygen and elevation in the partial pressure of carbon dioxide levels, as well as a chronic elevation of bicarbonate signifying a compensatory metabolic alkalosis for the respiratory acidosis. Lastly in this disease there remains a great need to thoroughly characterize the partitioned chest wall mechanics and work performed by the respiratory muscles during spontaneous breathing.

Based on somatic growth of the rabbit compared to the human, a 3 week old rabbit is equivalent to a 3 year old child, while a 28 week old rabbit is considered equivalent to a full grown adult. Pulmonary growth (alveolar septation) in the rabbit is thought to continue until skeletal maturity with the rate of growth decreasing monotonically from the first day of birth [14], similarly in humans recent evidence confirms that alveolarization continues throughout adolescence in children but at a markedly decreased rate after 2 years postnatal. The spinal deformity and FVC deficiency observed in severely deformed rabbits approaches that observed in patients with clinically significant EOS at maturity. Although longitudinal data on severe untreated EOS is rare Owange-Iraka et al. [7] retrospectively evaluated 30 patients with untreated idiopathic infantile scoliosis during adolescence and found an average 2-D scoliosis Cobb angle of 87° +/- 22.9° with an FVC of 53% +/- 15.6% expected, compared to a 99.8° +/- 16.3° 3-D composite maximum deformity angle and 71% +/- 10.1% expected FVC in our Severe rabbits. Additionally in this model we have shown here strong correlations between deformity with several other indicators of lung development and function. Untreated EOS is a known life threatening condition where progressive thoracic deformity leads to decline in respiratory function [6, 33–37]. The resulting low lung volumes lead to hypoxemia during sleep and eventually to cor pulmonale. Decreased chest wall distensability and diaphragm excursion limit the muscle work that the intercostals and diaphragm can perform during breathing. This is compensated by tachypnea, which contributes to increased fatigability, poor exercise tolerance and stunted statural growth. These patients have decreased FVC, which reflects decreased intra-thoracic volume, chest wall mobility and respiratory muscle function [7, 38]. Early intervention by expansion thoracoplasty has been shown to correct the thoracic deformity and increases the thoracic volume available for the lungs to grow [39]. However the clinical benefits of this treatment are inconsistent: reports of increased somatic growth [40] are offset by reports of decreased vital capacity [38] and no improvement in quality of life [41]. The present data forms a basis for the further controlled evaluation of expansion thoracoplasty treatment strategies and their potential to improve lung development and growth.

## Conclusions

We have created a rabbit model of early onset thoracic deformity to evaluate its influence on the growth, structure and function of the spine, thorax and lungs. Early progressive thoracic deformity during the phase of postnatal alveolarization induces pulmonary hypoplasia characterized by reduced lung growth, respiratory compliance, and abnormal ventilation characterized by diminished pulmonary reserves. The extent of deficiency in lung growth and function at adulthood was strongly related to the severity of the thoracic deformity present when the rabbit was young (equivalent to a 5 year old child), and thus confidence in expected growth and functional deficiency within this model has been quantified based on the thoracic geometry at youth. Early treatment of spinal deformity in children may prevent pulmonary complications in adulthood and this disease model will be useful as a platform to evaluate treatment effectiveness.

## Supporting Information

S1 Appendix Maximal deformity angle. (DOCX)

S1 Fig Spine standard projections. (TIF)

S2 Fig Spine maximal deformity projection. (TIF)

S3 Fig Diagram of the A-P projection of the spine. (TIF)
