# Supplementary material for: Extent of Spine Deformity Predicts Lung Growth and Function in Rabbit Model of Early Onset Scoliosis
Source: PLoS One. 2015 Aug 28;10(8):e0136941. doi: 10.1371/journal.pone.0136941 (PMC4552848; doi:10.1371/journal.pone.0136941)
Supplement: S1 Appendix — (DOCX) [file pone.0136941.s001.docx]

**Appendix**

**Extent of Spine Deformity Predicts Lung Growth and Function in Rabbit Model of Early Onset Scoliosis**

J. Casey Olson, Ayuko Takahashi, Michael P. Glotzbecker, Brian D. Snyder

**Maximal Deformity Angle**

The Cobb angle was defined by J.R. Cobb in 1948 (S1) to quantify sagittal alignment in scoliosis and has been extended for the measure of thoracic kyphosis. Because of its simplicity and reproducibility it remains the most common reported quantification of spinal deformity.

Cobb angle is the angle formed between a line drawn along the superior endplate of 1 vertebra above and 1 vertebrae below the curve of interest. In practice this is performed on an anterior to posterior (A-P) radiograph to quantify scoliosis (sagittal alignment) and endplates are chosen to provide the largest angle of deformity (Fig. S1-A ). Similarly the degree of kyphosis is quantified (although less frequently in practice) on a left to right (L-R) radiograph (Fig. S1-B) .

In this study we model the thoracic spine deformity as a single point of curvature in 3-dimensional space, such that a plane exists along the longitudinal axis on which the curved spine lies (Fig. S2-A) and when viewed on this perspective a maximal deformity angle, θ_M_ can be identified (Fig. S2-B). For example in the healthy spine with no scoliosis, the deformity plane is sagittal (viewed from the L-R projection) and the maximal deformity angle is the naturally occurring thoracic kyphosis Cobb angle.

The assumption that the curve lies on a single plane in space also requires that the curvature is proportionally similar when viewed from projections of any two perspectives. We also assume the apparent curvature in each projection (Fig. S1) is constant, such that an arc center with constant radius exists.

Thus from either the A-P or L-R projection a curved segment with height $h$, radius $R_{S,K}$, and alignment in the inferior endplate plane $(dx, dy)$ is defined (Fig. S3). Alignment of the spine in the inferior plane can thus be determined from the Cobb angles:$\tan\left( \frac{\theta_{S}}{2} \right)= \frac{dx}{h}$ and $\tan\left( \frac{\theta_{K}}{2} \right)= \frac{dy}{h}$. From this the orientation of the plane of deformity (clockwise from the sagittal plane), $\Phi=\tan^{-1}\left( \frac{\tan\left( \frac{\theta_{S}}{2} \right)}{\tan\left( \frac{\theta_{K}}{2} \right)} \right)=\tan^{-1} \left( \frac{dx}{dy} \right)$, and the Cobb angle in this plane, $\theta_{M}=2*\tan^{-1} \sqrt{\tan^{2} (\theta_{S}/2)+\tan^{2} (\theta_{K}/2})$, are defined.

The usefulness of this new composite deformity angle, θ_M_, can be evaluated by it's ability to predict pulmonary growth and function outcomes. Compared to the scoliosis (θ_S_) and kyphosis (θ_K_) angles θ_M_ had the strongest correlation in 6 of the 9 outcomes for which spinal curvature had a significant correlation (Table S1).

**Table S1. Deformity strength of correlation.**

| Predictors (28 wks)🡺 | **Θ_S_** | **Θ_K_** | **Θ_M_** |
| --- | --- | --- | --- |
| Outcomes (28 wks) 🡻 | **R^2^** | **R^2^** | **R^2^** |
| Body mass | 0.13 | **0.58***** | **0.46**** |
| Lung mass | **0.55***** | **0.79***** | **0.87***** |
| - Left lung | **0.28*** | **0.50**** | **0.49**** |
| - Right lung | **0.61***** | **0.83***** | **0.95***** |
| **Lung Volumes** |  |  |  |
| FRC | 0.01 | 0.01 | 0.00 |
| TLC | **0.57***** | **0.34*** | **0.52**** |
| - Left lung | 0.02 | 0.01 | 0.00 |
| - Right lung | **0.68***** | **0.54***** | **0.76***** |
| IC/TLC | 0.08 | 0.19 | 0.19 |
| - Left lung | 0.17 | **0.26*** | **0.31*** |
| - Right lung | 0.01 | 0.07 | 0.05 |
| **Thoracic Asymmetry** |  |  |  |
| R:L ratio | **0.55***** | **0.59***** | **0.78***** |
| TRA | **0.61***** | **0.65***** | **0.75***** |
| **Mechanics** |  |  |  |
| FVC | **0.66***** | **0.40*** | **0.64**** |
| Dyn. Resistance | 0.03 | 0.11 | 0.02 |
| Dyn. Elastance | 0.41 | **0.83***** | **0.93***** |
| Diaphragm S.A. | **0.56***** | **0.60***** | **0.71***** |

Comparison of the strength of correlation, by the coefficient of determination, between each of the measured spine deformity angles with final growth functional outcomes.

References

S1. Cobb JR. Outline for the study of scoliosis. In: Blount WP, editor. Instructional Course Lectures. Ann Arbor, MI: The American Academy of Orthopaedic Surgeons; 1948.

**S1 Fig. Spine standard projections.** (A) the A-P projection and (B) the L-R projection from CT scan of deformed rabbit thorax. The endplate lines and the Cobb angle, θ, are identified.

**S2 Fig. Spine maximal deformity projection.** The inferior-superior projection looking down the length of the spine (A) and the perspective perpendicular to the plane of deformity (B) from CT scan of deformed rabbit thorax. The curving spine lies on the plane of deformity as identified, and from this plane the maximal deformity angle, θ_M_, is determined.

**S3 Fig. Diagram of the A-P projection of the spine.** The red dashed line represents the curved segment of the spine, the solid blue lines are the lines drawn through the endplates perpendicular to the spine, each of these have the same length (ie. radius of curvature), h and dx mark the displacement of the spine perpendicular and tangent to the base plane respectively. A similar diagram is drawn for the kyphosis angle marked by a unique radius of curvature R_K_ and tangent displacement dy, but the same perpendicular displacement h.
