# Supplementary figures and images for: Extent of Spine Deformity Predicts Lung Growth and Function in Rabbit Model of Early Onset Scoliosis
Source: PLoS One. 2015 Aug 28;10(8):e0136941. doi: 10.1371/journal.pone.0136941 (PMC4552848; doi:10.1371/journal.pone.0136941)

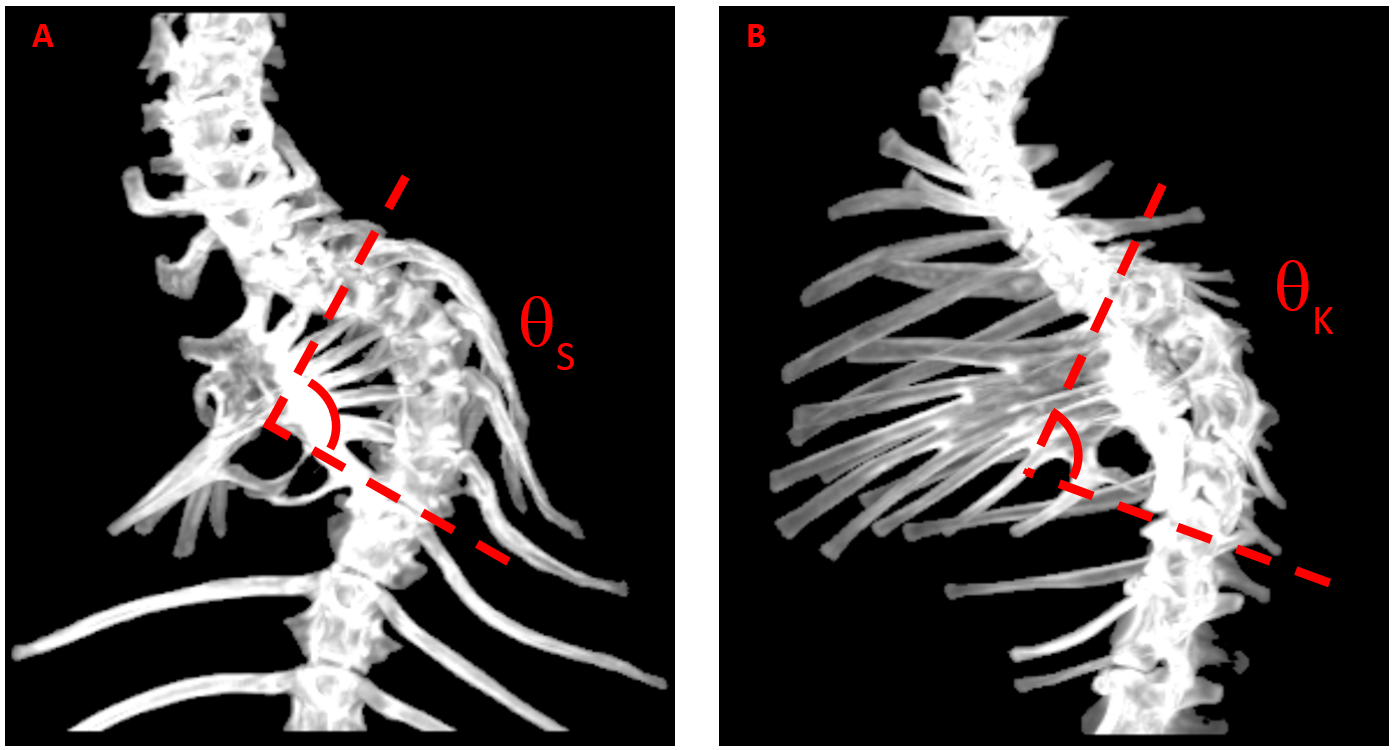

Supplement: S1 Fig — (TIF) [file pone.0136941.s002.tif]

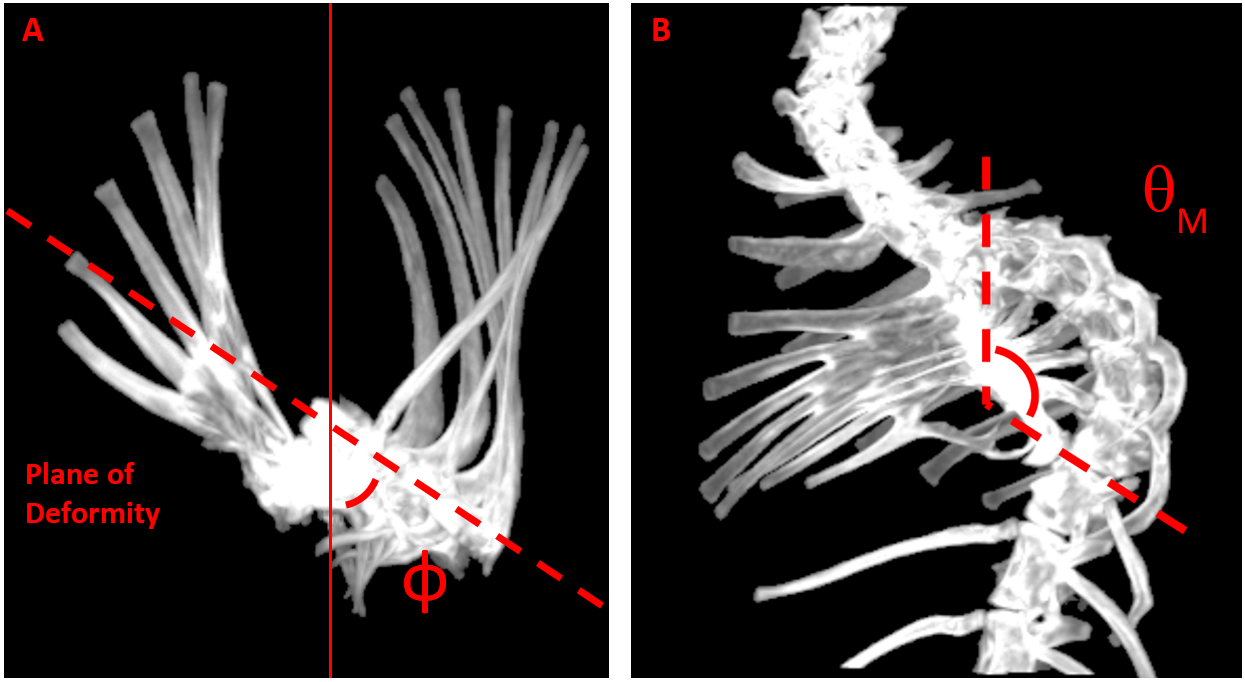

Supplement: S2 Fig — (TIF) [file pone.0136941.s003.tif]

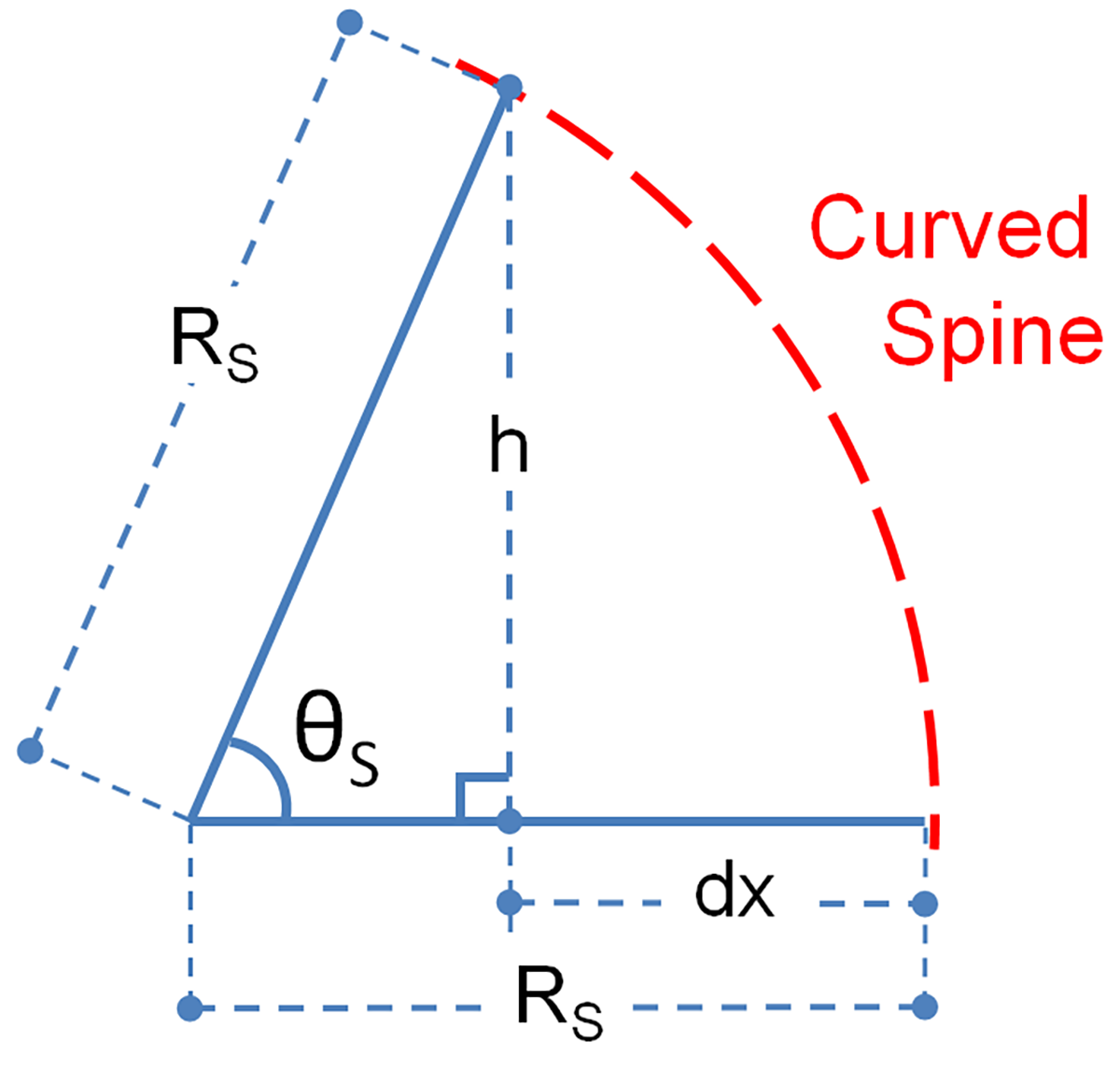

Supplement: S3 Fig — (TIF) [file pone.0136941.s004.tif]
